# Prevalence of Antibiotic Resistance Genes in Multidrug-Resistant *Enterobacteriaceae* on Portuguese Livestock Manure

**DOI:** 10.3390/antibiotics8010023

**Published:** 2019-03-13

**Authors:** Paula Amador, Ruben Fernandes, Cristina Prudêncio, Isabel Duarte

**Affiliations:** 1 Environment Department, Research Centre for Natural Resources, Environment and Society (CERNAS), College of Agriculture, Polytechnic of Coimbra, 3045-601 Coimbra, Portugal; iduarte@esac.pt; 2 Department Chemical Sciences and Biomolecules, School Allied Health Sciences, Polytechnic of Porto, 4200-072 Porto, Portugal; rfernandes@ess.ipp.pt (R.F.); cprudencio@estsp.ipp.pt (C.P.)

**Keywords:** antibiotic-resistance genes, multidrug resistance, livestock, animal excreta, environmental contaminants

## Abstract

The exposure of both crop fields and humans to antibiotic-resistant bacteria in animal excreta is an emergent concern of the One Health initiative. This study assessed the contamination of livestock manure from poultry, pig, dairy farms and slaughterhouses in Portugal with resistance determinants. The resistance profiles of 331 *Enterobacteriaceae* isolates to eight β-lactam (amoxicillin, cefoxitin, cefotaxime, cefpirome, aztreonam, ceftazidime, imipenem and meropenem) and to five non-β-lactam antibiotics (tetracycline (TET), trimethoprim/sulfamethoxazole (SXT), ciprofloxacin (CIP), chloramphenicol (CHL) and gentamicin) was investigated. Forty-nine integron and non-β-lactam resistance genes were also screened for. Rates of resistance to the 13 antibiotics ranged from 80.8% to 0.6%. Multidrug resistance (MDR) rates were highest in pig farm samples (79%). Thirty different integron and resistance genes were identified. These were mainly associated with resistance to CHL (*cat*I and *cat*II), CIP (mainly, *qnr*S, *qnr*B and *oqx*), TET (mainly *tet*(A) and *tet*(M)) and SXT (mostly *dfr*Ia group and *sul*3). In MDR isolates, integron presence and non-β-lactam resistance to TET, SXT and CHL were positively correlated. Overall, a high prevalence of MDR *Enterobacteriaceae* was found in livestock manure. The high gene diversity for antibiotic resistance identified in this study highlights the risk of MDR spread within the environment through manure use.

## 1. Introduction

The active ingredients of antibiotics (AB) legally prescribed in Portugal for veterinarian use in livestock prophylactic and metaphylactic treatments, amounted to 179,832 tons in 2013 [1]. Up to 90% of the ingested doses may be excreted unmodified or partially metabolised through urine and faeces [2]. Consequently, the excreta of AB-treated livestock became important reservoirs of AB residues, antibiotic-resistant bacteria (ARB) and antibiotic-resistance genes (ARGs) [3], where horizontal gene transference plays an essential role in the acquisition, spreading and assembly of various ARGs. Although integrons are not considered genetic elements per se, their location on plasmids and transposons enables gene transmission in an inter- and intra-species manner in a single event. For this reason, they are increasingly reported worldwide, especially among *Enterobacteriaceae* [4,5]. Large volumes of excreta enriched with *Enterobacteriaceae* end up as manure, slurry and wastewater, potentially vectoring these emergent contaminants. As such, they pose significant environmental concerns.

The slurry and the manure of intensively reared animals, composted or raw, are generally spread into soil for fertilisation; however, animal manure and wastewater are regarded as important potential AB-resistance reservoirs. Therefore, this reuse practice can lead to an increase of those pollutants in soil [6,7] and in endophytic bacteria of crops grown in manured soil [8]. The excreta ARB and ARGs can be spread over longer distances through other routes, such as anemophily [9], entomophily [10] or water run-off from farms and rural settlements, posing serious health problems. Despite the awareness about these spread routes, there are no available cost-effective tools to control the contaminants in municipal wastewater treatment plants (WWTPs) and livestock manure [11]. 

After recognising the importance of these emergent concerning contaminants, countries are developing their own national strategies to implement the Global Action Plan on antimicrobial resistance [12,13]. Moreover, the recent awareness that the health of humans and animals are inseparably interconnected with their environment led to an integrated One Health approach, particularly focusing on food safety, zoonosis surveillance and ARB control [14]. Dependence on livestock animals is considered one of the most critical pathogens associated risk factors to human given the transmission of AB resistance that occurs through consumption of contaminated animal products. *Enterobacteriaceae* is the major family associated with AB resistance and this is particularly relevant to this issue [15]. 

Understanding the diversity and the distribution of ARB and ARGs at a farm level is of great importance in order to identify the genetic background of this problem and, thus, educate and inform farm management. This study aimed to assess the level of contamination of livestock enterprises and the meat industry, in the central region of Portugal, through identification of multidrug resistant (MDR) isolates in manure. The analysis focused on the ARB and ARGs in resistant *Enterobacteriaceae*.

## 2. Materials and Methods

### 2.1. Study Area

**Figure 1:**
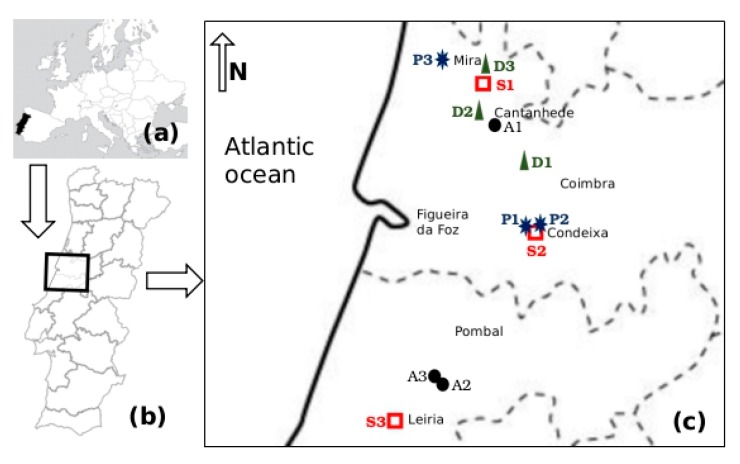
Location of sampling sites depicted on (**a**) a map of Europe (approximate scale 1:100,000,000, (**b**) a map of Portugal (approximate scale 1:10,000,000) and (**c**) a map of Coimbra and Leiria administrative regions (dashed lines) (approximate scale 1:1,000,000). Enterprise types are represented as follows: 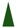 dairy cattle farm; 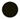 poultry farm; 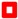 slaughterhouse; 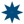 pig farm.

The study was conducted in three farm types and slaughterhouses representative of the livestock production sector in the central region of Portugal. The 12 selected small-scale enterprises included intensive raising of dairy cattle (D1, D2 and D3), poultry (A1, A2 and A3), pig (P1, P2 and P3) and three slaughterhouse companies (S1, S2 and S3). These sampling sites are located in the Coimbra and Leiria administrative regions, within Lis, Mondego and Vouga watersheds, at altitudes between 100 m and 200 m, higher than the flat alluvial river valleys (Figure 1). 

As per information obtained from the farms’ personnel, all animals had adequate veterinary monitoring and a few, when necessary, received medical treatments. The manure and slurry generated in these farms were either composted or directly used as raw soil fertilizers in the animal feed crop fields of each enterprise. According to the slaughterhouse personnel, all animals were subjected to the required withdrawal time as per public health safety regulations and the defined holding time before slaughter was followed. Each slaughterhouse had its own protocol for treating wastewater. 

### 2.2. Sampling

Between March 2016 and March 2017, three samples were collected per site. Dairy cows or swine manure samples were collected from open tanks with different maturation ages, whereas poultry manure from was collected from waste pools.

In the slaughterhouses, samples were collected from reservoirs holding the wastewaters generated by the daily slaughter of animals. The number of animals slaughtered daily is presented in Table 1.

Samples were collected in sterile plastic bottles or bags and maintained at 4 °C until the microbiological processing, which was always carried out within 4 h after sampling. In the laboratory, the three samples from each site were vigorously mixed and processed conjointly.

### 2.3. Microbiological Analysis and Phenotypic Characterisation

The enumeration, detection and identification of bacteria was carried out according to protocols previously described [16]. Decimal dilutions of the samples were prepared in sterile saline 0.9% NaCl. From each dilution, 100 mL were filtered through cellulose membranes of 0.45 µm (Millipore, Bedford, MA, USA) and the filters placed on the surface of selective medium for *Enterobacteriaceae* VRBG (Violet Red Bile Glucose) agar (Oxoid, Hampshire, England). The isolates were counted after overnight aerobic incubation at 37 °C. The colonies with different morphotypes were selected, picked out three times and their purity further confirmed microscopically. Species identification was performed according to the manufacturer’s recommendations by the standard API 20E galleries (BioMerieux, Marcy l’Etoile, Lyon, France).

Thirteen AB were selected for the phenotypic characterisation the isolates. These were chosen to represent the main AB classes used in human medicine and livestock production in Portugal, namely: amoxicillin/clavulanic acid combination (AMC) 30 μg/10 μg, respectively; ceftazidime (CAZ) 30 μg; cefotaxime (CTX) 30 μg; cefpirome (CPO) 30 μg; aztreonam (ATM) 30 μg; cefoxitin (FOX) 30 μg; imipenem (IPM) 10 μg; meropenem (MEM) 10 μg; chloramphenicol (CHL) 30 μg; gentamicin (GEN) 10 μg; ciprofloxacin (CIP) 5 μg; trimethoprim/sulfamethoxazol (SXT) combination (1:19) and tetracycline (TET) 30 μg. The disk diffusion Kirby-Bauer method was performed in agreement with the guidelines for antimicrobial susceptibility tests defined by the Clinical Laboratory Standards Institute [17], using *Escherichia coli* ATCC25922 (Liofilchem S.R.L., Roseto degli Abruzzi, Italy), *E. coli* J53-Az^R^ (provided by Instituto de Ciências Biomédicas Abel Salazar, Portugal) and *E. coli* HB101-Stp^R^–(Bio-Rad Laboratories Lda, Lisbon, Portugal) as quality controls, Mueller-Hinton agar and AB disks from Oxoid (Hampshire, England). The isolates with a resistance phenotype against three or more structurally unrelated antimicrobial agents were defined as multidrug resistant (MDR) [18]. 

### 2.4. Multiplex PCR (Polymerase Chain Reaction) for Genes Detection

The screening for resistance genes was focused on a subset of isolates chosen according to their phenotypic profile of resistance. The presence of the most frequent *Enterobacteriaceae* resistance genes in these isolates was determined by different multiplex PCR assays. Total DNA of these isolates was extracted as described by Amador et al [19] and 2 μL of each was subjected to multiplex PCR in a 25 μL reaction mixture containing 1× PCR buffer (200 mM Tris-HCl, pH 8.4, 500 mM KCl) and, according to the target gene, a variable concentration of primers (Table S1), MgCl_2_, dNTPs and 0.5 U of Taq DNA polymerase (Invitrogen, Thermo Fisher Scientific, Carlsbad, California, USA) (Table S2)**.** The PCR conditions described by referenced authors in Table S1 were modified as specified in Table S2.

The chloramphenicol-resistance genes were detected by a single multiplex PCR with four forward primers and one reverse primer to target four genes, namely, *cat*I, *cat*II, *cat*III and *cat*IV. The detection of *sul*1, *sul*2 and *sul*3 genes, that confer resistance to sulphonamide, was performed by two multiplex PCR assays given the different annealing temperature for *sul*3 gene amplification (Table S2). 

The search for 14 genes involved in three tetracycline resistance mechanisms, namely efflux pump, ribosomal protection and tetracycline enzymatic alteration, was performed by two multiplex PCRs, one targeting genes *tet*(A), *tet*(E), *tet*(G), *tet*(K), *tet*(L), *tet*(M), *tet*(O) and *tet*(S) and the other *tet*(B), *tet*(C), *tet*(D), *tet*(Q), *tet*A(P) and *tet*(X). 

For the detection of genes conferring resistance to trimethoprim, four distinct multiplex PCRs were performed according to the *dfr* gene group. The first PCR was used to detect genes belonging to groups Ia, (*dfr*A1, *dfr*A15, *dfr*A15b, *dfr*A16, *dfr*A16b, *dfr*A28), Ib (*dfr*A8) and Ic (*dfr*A12, *dfr*A13, *dfr*A21, *dfr*A22); the second for the group genes IIa (*dfr*A5, *dfr*A14, *dfr*A25, *dfr*A27), IIb (*dfr*A7, *dfr*A17) and IIc (*dfr*A3b); the third for groups III and IV, namely, IIIa (*dfr*A3), IIIb (*dfr*A10), IIIc (*dfr*A26), IVa (*dfr*A6), IVb (*dfr*A24), IVc (*dfr*A23); and the fourth for the groups Va (*dfr*B1, *dfr*B2, *dfr*B3, *dfr*B4, *dfr*B5, *dfr*B6), Vb (*dfr*A9), Vc (*dfr*A19) and Vd (*dfr*A20) [20].

Regarding plasmid-mediated quinolone resistance, nine genes responsible for conferring three different resistance mechanisms were analysed, namely, DNA gyrase protection from the action of the quinolones (*qnr*A, *qnr*B, *qnr*S, *qnr*C and *qnr*D), AB acetylation (*aac*(6’)-Ib-cr) and efflux pumps production (*qep*A, *oqx*A, *oqx*B). For this purpose, four different multiplex PCR assays were performed according to the annealing temperature (Table S2); one for the *qnr*A, *qnr*B and *aac*(6’)-Ib-cr; one for *qnr*D, *qnr*C and *qep*A; one for *qnr*S and *oqx*A; and a last one for *oqx*B gene.

To determine whether the ARGs were putatively disseminated via mobile genetic elements, three integrons classes, known to circulate in food-animal *Enterobacteriaceae*, were searched among the MDR isolates under study. Classes 1 and 2 of these genes were chosen for being most frequent among *Enterobacteriaceae*, whereas class 3 was chosen for being presently considered an emergent [21]. For this reason, a PCR was performed to detect *intI*1, *intI*2 and *intI*3 genes. 

Multiplex PCRs were performed in a thermocycler (iCycler, Bio-Rad, Thermal Cycler, Hercules, CA, USA) and the amplification products obtained were separated by electrophoresis on a 1% agarose gel (BioRad), stained with ethidium bromide (125 µg/mL) and visualized under a UV transillumination (Vilber Lourmat, Marne-la-Vallé, France).

## 3. Results and Discussion

MDR information is crucial to support environmental and public health mitigation actions. Environmental systems have very complex microbiome communities, which are difficult to study due to the inability to artificially culture the vast majority of the species present in a given sample. Metagenomic approaches have overcome this difficulty, revealing the vast genetic diversity of the collective molecular signature of the samples’ resistomes. However, these molecular methods alone do not allow attributing bacterial ARG to a single organism and therefore cannot be used to recognise MDR. This study is based on an organismal approach, selecting the in vitro cultivable ARB, specifically with the *Enterobacteriaceae* family. Consequently, the isolates characterized are a slice of the sample bacteria: the AB resistant *Enterobacteriaceae.*

### 3.1. Microbiological Parameters

The higher enumeration of the *Enterobacteriaceae* was recorded in manure samples from poultry farms when compared to those of slaughterhouse, swine and dairy cows (four orders of magnitude smaller than poultry). The average temperature observed in the samples ranged between 9.4–24.0 °C. The pH values of poultry and pig manure samples were neutral, while dairy cows manure slightly alkaline. Slaughterhouse samples varied from neutral to acid (Table 1).

Out of the 331 isolates recovered from 12 sampling sites, grouped by four enterprise types, 90 were originated from poultry, 71 from dairy, 81 from pig and 89 from slaughterhouse samples. The *Enterobacteriaceae* species, detected, by decreasing order of prevalence, were: *E. coli*, unidentified isolates, *Citrobacter freundii*, *Raoultella ornithinolytica*, *Salmonella enterica*, *C. koseri*, *Enterobacter cloacae*, *Morganella morganii*, *C. braakii*, *E. hermannii*, *E. vulneris*, *Klebsiella oxytoca*, *Kluyvera* spp. A higher frequency of *E. coli* was registered in samples from slaughterhouses, pig and poultry farms, whereas *C. freundii* was more prevalent in dairy farm samples (Table 2).

### 3.2. Antibiotic Resistance Profile

**Figure 2:**
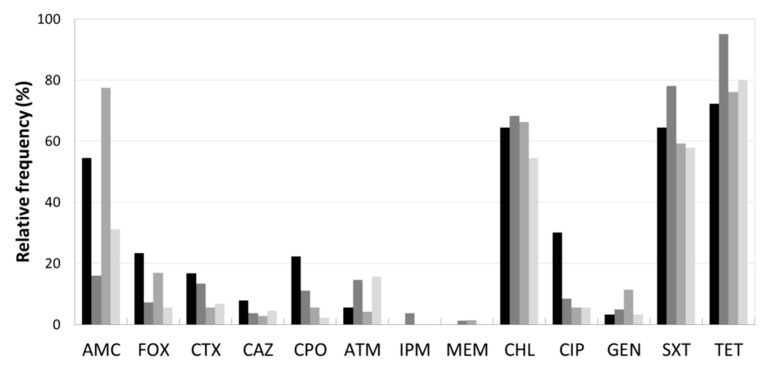
Antibiotic resistance of *Enterobacteriaceae* isolates obtained from 12 sampling sites. Relative frequency of resistance patterns exhibited by resistant and intermediate resistant isolates to 13 AB. Tested AB: eight β-lactams: amoxicillin/clavulanic acid (AMC), ceftazidime (CAZ), cefotaxime (CTX), cefpirome (CPO), aztreonam (ATM), cefoxitin (FOX), imipenem (IPM), meropenem (MEM) and non-β-lactams: chloramphenicol (CHL), tetracycline (TET), gentamicin (GEN), trimethoprim/sulfamethoxazol (SXT) and ciprofloxacin (CIP). Enterprise type: 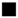 poultry, 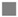 pig, 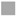 dairy, 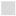 slaughterhouse.

The analysis of AB resistance profiles of the isolates under study revealed by decreasing frequency order, among the β-lactam group, AMC, FOX, CPO, CTX, ATM and CAZ. The carbapenems, IPM and MEM, had the lowest frequencies. With regards to the non-β-lactam group, TET, SXT and CHL revealed to be less effective, whereas CIP and GEN were the most effectives (Figure 2). These findings can be explained by the intensive and recurrent use of tetracyclines, sulphonamides, penicillins and 2nd, 3rd and 4th cephalosporins generation in livestock production in Portugal [1] and all over the world [22,23]. The last report of the national control plan of drug use in Portugal referred the tetracyclines (38.9%), penicillins (18.8%), quinolones (4.5%) and sulphonamides (3.4%), as the AB classes for veterinary use with higher sales [1]. Curiously, as chloramphenicol (CHL) was banned in livestock according to the European legislation [24], the low sales of amphenicols (0.7%), apparently not justify the high frequency of CHL resistant isolates.

The AB resistance patterns per enterprise type revealed that the isolates from poultry farms had the highest resistance rates to TET, SXT, CHL and AMC (Figure 2). These results are in agreement with reports from: (i) Ghana, with high resistance frequencies to TET (88.9%), sulphonamide (75.0%), ampicillin (69.4%) and trimethoprim (66.7%) [25]; (ii) Madagascar broil farms with 97.6% to TET [26]; (iii) Chinese carcasses with ampicillin (98.9%), CHL (92.2%), TET (78.9%) [27]; and (iv) Portuguese healthy chickens with TET (70%), ampicillin (63%) and CIP (49%), however, with lower frequencies for SXT (33%), CHL (12%) and AMC (18%) [28]. It is noteworthy a higher frequency of poultry isolates resistant to CIP (30.0%) compared with those from the other enterprise types. An increasing resistance to this AB in poultry farming have been reported, for example from Canada [29], China [30], Brazil [31] and India [32]. This raise of resistance to CIP might be due to the increasing administration of quinolones to treat avian infections [21].

No isolate resisted to MEM and IMP (Figure 2), which agrees with many other studies carried out in livestock environments, showing only sporadic reports of isolates resistant to carbapenemases [28,33].

Regarding pig farms, the resistance rates of the isolates were higher for TET, SXT and CHL, followed by the β-lactams AMC, ATM and CTX (Figure 2). The lower resistance frequencies to carbapenems, IPM and MEM were not surprising, as previously mentioned. Similar results were published from China with over 60% for TET and CHL and no resistances to carbapenem (IMP) [34]. In the same country, another study revealed identical results towards carbapenems but all isolates were resistant to TET, ampicillin, CAZ and CTX. However, contrary to our results, a lower incidence for CHL (26.3%) and SXT (52.6%) were detected [35]. The association among the resistance phenotypes ampicillin–doxycycline–TET–SXT was reported as the predominant in poultry and swine production in China [36].

Among dairy farm isolates, higher resistance prevalence was observed to AMC, followed by TET, CHL and SXT. A single isolate resistant to carbapenem, MEM was found (Figure 2). These results are in agreement with many studies carried out in dairy farms, which usually report high TET and β-lactamic group resistances [37] and low or null carbapenem resistance. However, disagreeing with most published results, SXT resistances were lower than to CHL. 

The AB resistance patterns (TET, SXT, CHL and AMC) of slaughterhouses isolates were similar to those of the other three enterprise types. These are unsurprising results, once the majority of the animals slaughtered in the abattoirs in this region, are locally raised cattle and pigs. Moreover, these results are consistent with many other studies carried out in agricultural environments. For instance, a Portuguese study on *S. enterica* and *E. coli* strains isolated from pork and beef products revealed similar resistances to TET, sulfamethoxazole and ampicillin [38]. In addition, a study in swine faecal samples obtained from Spanish slaughterhouses, showed the following resistance percentages: TET (90%), AMC (78%); SXT (67.5%); CHL (26%); GEN (6%) and CTX (0%) [39].

The comprehensive analysis of AB resistances rates by enterprise type revealed differences between poultry and pig isolates compared to those of dairy cattle. This seems consistent with other results [21] also reporting a lower prevalence of resistant *E. coli* in cattle than in pig and poultry. The highest rates of resistance to all the AB were found among the *E. coli* and the unidentified *Enterobacteriaceae* isolates. Worth noting was the high resistance to CTX in *Citrobacter freundii* and to MEM in *Kluyvera* spp. (Figure S1).

**Figure 3:**
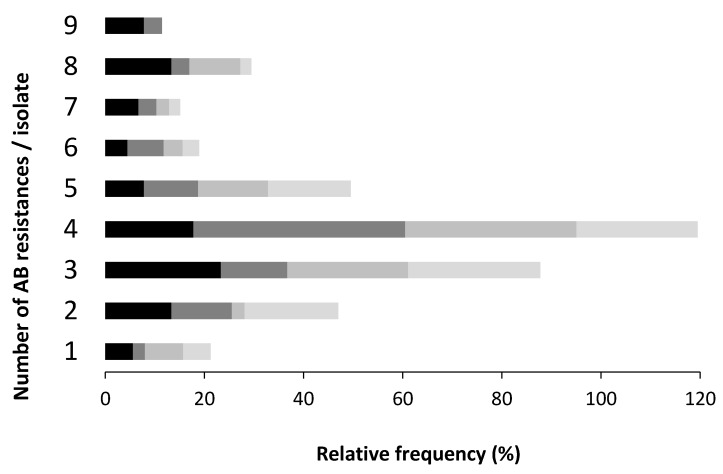
Relative frequency of the number of antibiotics to which each isolate resists. Enterprise type: 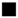 poultry, 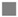 pig, 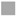 dairy, 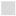 slaughterhouse.

The majority of the isolates under study resisted simultaneously to three or four AB, regardless the sample origin. Poultry farm samples had the highest rate of resistant isolates to more than seven AB, when compared with slaughterhouse samples, where a higher rate of isolates simultaneously resistant to a lower number of AB was recorded (Figure 3).

The MDR rates observed in this study, poultry (71%), pig (79%), dairy cattle (69%) and slaughterhouse (63%), were higher if compared with other three Portuguese studies. One with pork samples, (60.7%) [40], other with chicken carcasses samples (56%) [28] and the third one with faecal and tissues samples of chickens and pigs (over 50%) [41]. The increasing trend of the AB co-resistant phenotypes numbers in humans and animals is a consequence over time of the intensive broad spectrum AB use and the introduction of newer compounds in human and veterinary medicine. This increase of MDR isolates, all over the world, can be explained by the mobile genetic elements in bacteria isolated from facilities farms, including WWTP, farm surrounding air, faeces, carcasses and organs of the animals, identified in numerous studies [21]. 

### 3.3. Screening of AB Resistance and Integrons Genes

Based on phenotypically characterised isolates, 70 MDR (22, 19, 13 and 16 isolated from poultry, pig, dairy and slaughterhouse samples, respectively) were selected for molecular characterisation (Table 2 and Table 3).

#### 3.3.1. Chloramphenicol

All the isolates harbouring CHL resistance genes revealed an according phenotypic resistance profile (Table 2). However, in 61.4% (43/70) of those isolates no *cat* genes were detected. These results may be explained by the CHL resistance mechanisms, which are not exclusively determined by the *cat* genes family (I, II, III and IV). Although, the plasmid-mediated chloramphenicol acetyltransferase (*cat*) gene is the most common mechanism, other non-enzymatic resistance plasmidic genes, *cml*A and *flo*R, encoding efflux pumps, have also been identified in *Enterobacteriaceae*. The latter gene encodes a florfenicol/chloramphenicol transporter that confers resistance to both AB. A less common resistance mechanism involves mutations in 50S ribosomal subunit [42,43].

No CHL resistant isolates harboured genes *cat*III and *cat*IV and those which had a gene, had either *cat*I or *cat*II (Table 3). The highest frequencies of *cat* genes were detected in samples from poultry and dairy cattle farms. The gene *cat*I was more prevalent (21.4%) than *cat*II (2.9%), the latter only represented by two *M. morganii* isolates from dairy samples (Table 3). Similarly, a study on MDR *E. coli* from Irish cattle and the farm environment, reported 9% CHL resistant isolates, mainly mediated by *cat*I and *flo*R [42]. Other reports also reveal that *cat*I gene together with *cat*III gene is the most widely distributed, being *cat*II less frequent [44]. However, a Korean study on fish pathogens isolates detected the *cat*II gene as the most frequent, although *cat*IV was also found in some isolates [45].

The high resistance to CHL found in this and other studies may have several explanatory hypotheses. Assuming that this AB is no longer used in human and veterinary medicine due to its proven chronic toxicity, the bacteria continue to resist because the determinant genes (often integrated in gene cassettes inserted in genetic elements, including integrons) could be transferred among them, even without high selective drug pressure. Another explanation is the licensed use of florfenicol for treatment of respiratory infections in cattle and pigs (Regulation (EEC) No 2377/90). This AB is a derivative of CHL, which shares one gene that determines the resistance mechanism [21].

#### 3.3.2. Quinolones

The detection of the genes conferring resistance to the quinolones class, showed a higher incidence among isolates of poultry farms (Table 2). Quinolones, such as difloxacin, enrofloxacin, danofloxacin, marbofloxacin, pradofloxacin represent 4.5% of the AB sales, mainly for the avian sector in Portugal [1]. Likewise, ciprofloxacin (CIP), levofloxacin, norfloxacin, ofloxacin are widely used clinically to treat avian colibacillosis in many other countries [30]. The intensification of this AB class usage might explain the higher relative frequencies of CIP resistance in poultry isolates than others, sampled in other enterprise types (Figure 2). These quinolones class resistances might difficult the future therapy in this livestock sector.

No *qnr*A, *oqx*A, *aac*(6’)–Ib-cr and *qep*A genes were detected in any isolate. The *qnr*S gene was the most prevalent, mainly in the assayed poultry isolates (Table 3; Table 4), which is consistent with previous works carried out worldwide. An Italian work showed a higher frequency of the *qnr*S1 and *qnr*B19 genes for poultry isolates, respectively but no genes of *qnr*C, *qnr*D, *qep*A and *aac*(6’)-Ib-cr [46]. Other study involving *E. coli* isolates from chicken farms and turkeys at slaughterhouses in the Czech Republic, showed through screening of several genes, namely, *qnr*B1, *qnr*B4, *qnr*B8, *qnr*B10, *qnr*B19, *qnr*D, *qnr*S1, qnrS2, *aac*(6’)-Ib-cr and *oqx*AB, the same trend of results, a higher prevalence of *qnr*S1 followed by *qnr*B19 [47]. Another Chinese study, investigating the quinolones resistance in *E. coli* isolated from septicaemic broilers, namely, *qnr*A, *qnr*B, *qnr*S and *aac*(6’)-Ib-cr, recorded the highest prevalence of the gene *aac*(6’)-Ib-cr (36.0%), followed by *qnr*S (8.1%), *qnr*B (0.9%) and *qnr*A (0%) [30]. Conversely, in Nigerian poultry faecal samples no isolates containing the *qnr*S gene were registered, being *oqx*B the most prevalent [48].

Considering the pig farm isolates, our data revealed a higher prevalence for the *qnr*B gene, followed by the same prevalence for *qnr*S and *oqx*B, with 2 isolates each (Table 3). A study on sewage and soil adjacent to swine feedlots, showed a higher frequency for the *qnr*D, *qep*A and *oqx*B genes, whereas *qnr*S and *oqx*A were only present in the samples of residual waters [49].

#### 3.3.3. Tetracycline

The screening of the 14 most frequent *tet* genes conferring resistance to TET revealed that genotypes are consistent with the phenotypes observed, excepting for three isolates, from pig and dairy farms (Table 2 and Table 3). The TET resistance in those isolates without *tet* genes, might be due to other resistance genes, not targeted in this study. There are at least 47 distinct genes identified, responsible for four main mechanisms by which the bacteria acquire resistance to tetracyclines [50].

Additionally, no isolates harboured the following genes: *tet*(D) and *tet*(G), responsible for the efflux pump resistance mechanism; *tet*(S) and *tet*(Q), responsible for the mechanism of ribosomal protection and *tet*(X), for the degradation of AB (Table 3). The most incident genes were *tet*(A) and *tet*(M) (Table 3), encoding an efflux pump and providing ribosomal protection, respectively. All the other surveyed genes had frequencies below or equal 10%. The gene *tet*(A) prevailed in isolates of all the four enterprise types; however, the second most frequent gene varied according to the sample source: *tet*(L) in poultry farms, *tet*(E) in dairy farms and *tet*(M) in pig farms and slaughterhouses. The prevalence of *tet*(A) gene in this study is in agreement with the dominant *tet* gene in different animal and environmental samples reported worldwide in a myriad of studies [42,51,52,53,54]. 

The isolates from poultry farms harboured the highest diversity of different *tet* genes (9/14), followed by slaughterhouses isolates with (7/14), conversely to those from dairy and pig farms, which showed lower genes diversity. The *tet*(C) gene was unique from poultry isolates group. Regarding the number of different *tet* genes per isolate, 14.3%, 45.7%, 27.1% and 7.1% of them contained, zero, one, two and three, respectively (Table 2). It should also be noted that one isolate of *E. cloacae* and one of *E. coli* from the poultry farms presented five different genes, while two other isolates, a *S. enterica* from laying hens samples and an *E. coli*, from the slaughterhouse, presented four genes. The analysis by sample source showed that the poultry farm isolates had the higher number of resistant isolates containing more *tet* gene types simultaneously. Identical results were reported in a study carried out in swine sewage ponds, where the isolates most often harbour multiple *tet* genes [55].

#### 3.3.4. Sulphonamide

The most prevalent resistance genes to the sulphonamide class were *sul*3, followed by *sul*1 and at last *sul*2 (Table 3); however, this ranking varies according to the sample source. For instance, among the poultry farm isolates genes *sul*1 and *sul*3 were more frequent than *sul*2. Curiously, the isolates from dairy farms presented a higher incidence of *sul*1, whereas *sul*3 prevailed in slaughterhouses isolates and *sul*2 in those from pig farms. These genes prevalence can vary greatly with the matrixes, geographic region, as well as the species.

Our results are partially in accordance with two studies carried out in Denmark on isolates of *E. coli* resistant to sulphonamide obtained from swine faeces. The first work presented the highest frequency for the *sul*1 gene (55%), followed by *sul*2 (50%) and finally *sul*3 (11%) [56], whereas the second work reported *sul*2 gene, as the most prevalent (65%) [57]. On the other hand, a Portuguese study on resistances of *E. coli* isolated from carcasses and internal organs of healthy chickens from intensive farms detected 37 different resistance genes and the most common were *tet*(A) (72%) and *sul*1 (47%) [28]. A study in Cambodia on sulfamethoxazole-resistant isolates obtained from faecal samples of healthy chickens found a higher prevalence of the *sul*2 gene in *E. coli* (56%) and in *Salmonella* Corvallis (53%). However, among the *Salmonella* Albany the most prevalent gene was *sul*1 (54%) [58].

The analysis by isolates shows that 22.9% did not have a *sul* gene, 30.0% harboured one type of *sul* gene, 34.3% contained simultaneously two and 12.9% three *sul* genes (Table 2). It should be noted that the latter isolates were more prevalent in poultry and pig farm samples, which corroborates the idea that there is a higher AB selective pressure in these two livestock intensive raising systems.

#### 3.3.5. Trimethoprim

Regarding the *dfr* genes detected in this study, a higher prevalence of the *dfr*Ia group was found as a general trend in all enterprise type samples; however, being most prevalent in those of poultry farms (Table 3). Genes of *dfr*Ic, *dfr*IIIb, *dfr*IIb groups were recorded but *dfr*IV and *dfr*V were not identified. Moreover, the *dfr*IIc group genes had the lowest detection rate, in only an isolate from a dairy farm sample. The isolates of poultry and dairy farms showed the highest diversity in this gene family. These results are in accordance with those of a Lithuanian study about the prevalence of *dfr* genes on *E. coli* isolates from human and animal clinical samples, concluding that 92% contained at least one of the six genes, *dfr*A1, *dfr*A5, *dfr*A8, *dfr*A12, *dfr*A14 and *dfr*A17 [20]. This gene nomenclature corresponds in our study to the genes of the group *dfr*Ia, *dfr*IIa, *dfr*Ib, *dfr*Ic, *dfr*IIa, *dfr*IIb, respectively). The genes *dfr*A1 (*dfr*Ia group) and *dfr*A17 (*dfr*IIb group) were more frequently found in clinical isolates, while the *dfr*A1 (*dfr*Ia group) and *dfr*A14 (*dfr*IIa group) genes dominated in isolates of animal origin. The genes *dfr*A5 (*dfr*IIa group), *dfr*A12 (*dfr*Ic group) and *dfr*A8 (*dfr*Ib group) were detected at lower frequencies.

For the analysis of the genes involved in SXT resistance it is required to consider simultaneously the genes *sul* and *dfr*, since they act synergistically to confer resistance to an association of two antimicrobials, the sulfamethoxazole and the trimethoprim, both inhibitors of the biosynthetic pathway of folic acid. Resistance to both AB occurs by the acquisition of genes encoding enzymes that have no affinity for binding to these antimicrobial compounds, inactivating them [43].

All isolates listed in Table 2 with at least one type of gene from each family, *sul* or *dfr* exhibited SXT resistance, as for example some SXT resistant isolates without *dfr* genes but with one or more *sul* genes. This occurrence may be explained, as previously stated, by the presence of other genes that were not searched in this study, since there are more than 30 different known genes of trimethoprim resistance [20]. On the contrary, the phenotype of SXT resistant isolates lacking the *sul* genes might be explained by chromosomal mutations, resulting in molecular variants providing this resistance [43], even in absence of the resistance genes *sul*1, *sul*2 and *sul*3.

#### 3.3.6. Integrons

The analysis of the AB resistance profiles reveals their clear MDR phenotype (Table 2). Those isolates with co-resistant phenotypes showed that the underlying resistance determinants of the non-β-lactams are based on three or more gene groups: tetracycline (*tet*(A) and *tet*(M)), sulfamethoxazole (*sul*3 and *sul*1), trimethoprim (*dfr*A1-like) and CHL (*cat*I) (Table 4). Although it was not the scope of this work, the information on the Extended Spectrum Beta-Lactamases (ESBL) genotyping of these isolates would have been interesting, as these enzymes are often associated with the MDR [59]. Nevertheless, our results show the prevalence of a common resistance association pattern of several gene classes, which are known to be specially spread and persistent in livestock environments [21]. The widespread dissemination of the same MDR phenotypes mediating AB resistance was also reported by the European surveys on healthy food animals [22,60]. Other studies also show a predominant association among ampicillin-tetracycline-sulfamethoxazole/trimethoprim in Chinese poultry and pig farming [36].

The search for three integrons classes among the 65 MDR isolates under study, revealing 25 integrons (38.5%) of classes 1 and 2 and none of class 3. This latter, result is expected, as it is not prevalent in *Enterobacteriaceae*. The integrase gene *IntI*2 (class 2), initially present in Tn7 transposon and derivatives, is more frequent than the class 3 integrons [61]. Moreover, no isolate harboured simultaneously two integrons classes. 

Class 1 and class 2, with 16 and 9 integrons, respectively were the most prevalent in isolates of all enterprise type samples (Table 3). These results are consistent with those of a Portuguese study conducted in poultry and pigs farms, having identified five isolates with class 1 of integrons and one isolate with class 2 [41]. Likewise, other US study on *Enterobacteriaceae* isolates obtained from fish, poultry, swine and cattle reported that 52% of them contained either class 1 or class 2 integrases, with the frequencies of 46% *intI*1, 6% *intI*2 and 5% *intI*1 plus *intI*2 [62]. Additionally, another study performed on MDR *K. pneumoniae* isolates from Iranian hospitals showed similar trends regarding the prevalence of the two integrons classes and a positive association between the class 1 integrons and MDR [63]. A Polish investigation on clinical samples from patients not undergoing AB therapy and from a WWTP detected 12.1% of the *E. coli* isolates with integrase genes, 10.9% of which with class 1, 1.4% with class 2 and 0% with class 3 [64].

Contrary to what was expected, the integrons were not prevalent among the isolates of poultry farm samples, which contradict the idea that the prevalence of integrons could reflect a strong AB pressure, selecting for those isolates with integrons [64]. In fact, the highest prevalence was recorded in pig farm isolates, with 11 integrons of both classes (Table 3). These frequency trends of the integrons classes are somewhat consistent with those found in *E. coli* isolated from samples of different livestock environments in Georgia reported for: (i) poultry isolates, *intI*1 (66%), *intI*2 (14%) and *intI*1 plus *intI*2 (11%); (ii) swine isolates, *intI*1 (86%), *intI*2 (0%) and *intI*1 plus *intI*2 (0%); and iii) beef cattle isolates, *intI*1 (75%), *intI*2 (23%) and *intI*1 plus *intI*2 (20%) [62].

Comparing the resistances to the non-β-lactamic AB with the presence of integrons in all the isolates under study, a positive relation between the SXT, CHL and TET resistant phenotype with the presence of integrons was observed. This relation is less evident with the quinolone CIP and aminoglycoside GEN (Table 4).

A Portuguese study on isolates from several polluted rivers, due to heavy domestic, industrial and agricultural influence, registered among the cefotaxime-resistant isolates, the prevalence of class 1 integrase (56.41% in those ESBL^+^) and the gene cassettes identified conferred resistance to β-lactams, trimethoprim and chloramphenicol [59]. The different sampling matrices may account to dissimilar results to ours, regarding CIP and GEN. However, several studies revealing the diversity of class 1 integrons and the gene cassettes in *Enterobacteriaceae*, indicate that the integrons generate new bindings of AB resistances, mainly in areas highly exposed to antimicrobial agents [65].

## 4. Conclusions

This study demonstrated a high prevalence of MDR *Enterobacteriaceae* and high resistance-gene diversity in livestock manure, possibly a consequence of intensive animal farming. Therefore, field amended with manure containing these biological contaminants can potentially contribute to an increased frequency of MDR bacteria in surrounding soil and aquatic ecosystems. As untreated manure is often used as a fertiliser of maize, rice and horticultural crops in agricultural fields of the Vouga, Lis and Mondego valleys, the common practices of nutrient and organic matter reuse for crop production has become a matter of concern, since the resistance of zoonotic bacteria should also be considered, as those contaminants may be transferred to livestock and humans through the consumption of raw crops fertilised with contaminated manure.

It should be important not only to reinforce the prudent, ethical and professional use of AB but also to improve appropriate treatments in the WWTP of the farms and slaughterhouses. Our findings provide an insight into the real impact of livestock farming manure to the environmental and public health problem of these contaminants in the Central Region of mainland Portugal.

## Figures and Tables

**Table 1:** Animal heads per sampling site, average microbiological and physical-chemical parameters of samples.

Characterisation of Sampling Sites			Characterisation of Samples		
Site Code	Enterprise Type	Animal Heads	*Enterobacteriaceae* (cfu/mL)	Temperature (°C)	pH
A1	Poultry	10,000 chicks Legorne/pavilion	1.4 × 10^7^	10.2	6.80
A2	Poultry	72,000 caged laying hens Legorne	6.9 × 10^9^	24.1	7.24
A3	Poultry	28,000 soil, cage-free laying hens Legorne	7.6 × 10^9^	24.0	7.06
D1	Dairy cattle	100 Holstein Friesian	1.7 × 10^5^	9.4	8.81
D2	Dairy cattle	30 Holstein Friesian	5.7 × 10^5^	14.6	7.46
D3	Dairy cattle	54 Holstein Friesian	2.4 × 10^5^	16.9	8.47
P1	Pig	48 breeding sow Large Write	3.4 × 10^5^	14.7	7.71
P2	Pig	700 breeding sow, 5300 piglets Large Write	5.0 × 10^5^	14.0	7.16
P3	Pig	1640 fattening pigs Large Write	1.7 × 10^6^	13.8	7.33
S1	Slaughterhouse	505 piglets, 62 cattle (daily abattoir)	1.0 × 10^6^	14.1	6.52
S2	Slaughterhouse	381 hogs, 740 cattle (daily abattoir)	1.7 × 10^6^	16.0	4.92
S3	Slaughterhouse	190 hogs, 40 cattle (daily abattoir)	1.1 × 10^9^	23.8	7.31

Temperature (°C)—determined by thermometry; pH—determined by potentiometry.

**Table 2:** List of AB resistance profile, AB resistance and integrons genes harboured per isolate species and sample origin.

Sample ^(a)^	*Species*	Resistance Phenotype ^(b)^													AB Resistance Genes					*int*I
		AMC	FOX	CTX	CAZ	CPO	ATM	IPM	MEM	CHL	CIP	GEN	SXT	TET	*cat*	*qnr*/*oqx*	*tet*	*sul*	*dfr*	
**Poultry**	*S. enterica*	x	x	x	x					x	x		x	x	-	-	A, M, L, K	1, 2, 3	Ia, IIb	-
	*Enterobacter cloacae*	x	x	x	x		x			x	x		x	x	I	D, S	A, E, B, K, O	1, 3	Ia	-
	*E. hermannii*	x	x		x		x			x	x	x	x	x	-	-	C	1, 2	Ic	-
	*C. freundii*	x	x	x			x			x	x		x	x	I	C, S	A	1, 2	Ia, IIb, IIIc	-
	*C. freundii*	x	x	x	x					x	x		x	x	I	S	A, L, K	1, 2	Ia, Ic, IIIa, IIIc	-
	*R. ornithinolytica*	x				x				x			x		-	nt	-	3	Ia	1
	*E. coli*	x	x	x	x	x	x	x					x	x	-	nt	A, C	1, 2, 3	Ia, Ib, IIIa	-
	*E. coli*	x	x							x			x	x	-	nt	A, E, C, M, L	2, 3	Ia, IIa	-
	*E. coli*	x	x			x		x		x			x	x	-	nt	A	1, 3	Ia, Ib	-
	*E. coli*	x	x		x	x		x		x			x	x	-	nt	A	1, 3	Ia, Ib, Ic, IIIa	-
	*E. coli*	x	x								x		x	x	-	nt	A	2	Ia, Ic, IIb	-
	*E. coli*	x			x	x		x		x			x	x	-	nt	A	3	Ia, Ib	-
	nt	x	x	x	x					x	x		x	x	I	S	A, M	1, 2, 3	Ia, IIb	-
	nt	x	x	x	x		x			x	x		x	x	I	S	A, A(P)	1, 3	Ia	-
	nt	x	x				x			x	x		x	x	-	C, S	A	1, 3	Ia	1
	nt	x	x	x			x			x	x		x	x	I	C, S	A	1, 2, 3	Ia	1
	nt	x	x							x	x		x	x	I	B, S	A	1, 3	-	I
	nt	x	x	x						x	x		x		I	B, C, D, S	-	1	Ia	1
	nt	x								x		x		x	-	nt	A, A(P), M	-	IIb	-
	nt	x	x			x				x			x	x	-	nt	A	-	-	-
	nt	x								x	x		x	x	-	B, S	A, L, K	3	-	-
	nt	x								x	x		x	x	-	B, S	A, L	1, 3	Ia, Ic, IIb	-
**Pig**	*S. enterica*	x		x						x			x	x	-	nt	A	-	Ia, Ic	2
	*K. oxytoca*	x	x								x		x	x	-	*oqx*B	A	1, 2, 3	Ic, IIa	-
	*R. ornithinolytica*	x	x	x	x			x		x				x	I	B	M	2, 3	-	-
	*Kluyvera* spp.	x	x		x		x	x	x	x			x	x	-	nt	A	2	Ia	2
	*E. coli*	x								x		x		x	-	nt	A, B	-	Ic	-
	*E. coli*	x			x		x	x		x			x	x	I	-	A	1, 2, 3	Ia, Ic	1
	*E. coli*	x					x	x		x			x	x	-	B	A	1, 2, 3	Ia	2
	*E. coli*	x			x		x	x		x				x	-	-	-	-	Ia, IIIb	-
	*E. coli*	x			x		x	x		x	x		x	x	I	-	B, M	3	Ia, IIa	2
	*E. coli*	x			x	x	x	x		x				x	-	B	A	-	Ia	-
	*E. coli*	x			x	x	x	x		x	x		x	x	-	B	A	-	Ia, IIIb	1
	*E. coli*	x	x	x	x			x	x	x			x	x	-	B	A	1, 2	Ia, Ic	-
	nt	x									x		x	x	-	*oqx*B	A, M	1, 2, 3	-	1
	nt	x		x					x	x			x	x	-	nt	-	-	-	2
	nt	x								x	x	x	x	x	-	B, S	A	2	IIIb	-
	nt	x		x					x	x			x	x	-	nt	A, M, K	2, 3	-	2
	nt	x	x							x	x		x	x	-	S	A, M	3	-	1
	nt	x			x		x	x		x			x	x	-	nt	A	2	IIIb	-
	nt	x			x		x	x		x			x	x	-	nt	A	2, 3	-	1
**Dairy**	*M. morganii*	x	x	x						x				x	II	nt	A, O	1, 2	-	-
	*M. morganii*	x	x	x	x					x			x	x	II	nt	A, K	1, 2	-	-
	*C. freundii*	x	x	x						x	x		x	x	I	S	A	1, 2	Ia	-
	*C. freundii*	x	x	x						x	x		x		I	C, S	-	1, 3	Ia	1
	*C. freundii*	x	x	x						x	x		x	x	I	C, S	A	1, 3	Ia	1
	*C. braakii*	x	x	x	x	x		x						x	-	nt	A	-	Ib	-
	*C. koseri*	x	x		x	x	x	x				x	x	x	-	nt	A, E	1, 3	Ib, IIIb	-
	*R. ornithinolytica*	x	x							x				x	-	nt	A, E	-	Ia, Ib	-
	*C. koseri*	x	x					x		x					-	nt	-	-	IIIc	-
	*E. coli*	x									x		x	x	-	nt	A	2	Ia, IIa	-
	*E. coli*	x	x				x			x			x	x	-	nt	A, E	1	IIc	-
	*E. coli*	x	x						x	x				x	-	nt	-	-	Ia	-
	nt	x	x	x						x			x	x	-	nt	M	3	IIIb, IIIc	-
**Slaughterhouse**	*E. coli*	x				x								x	-	nt	A(P), L	-	Ia, Ic	2
	*E. coli*	x						x			x		x	x	-	nt	O, M	3	Ia	-
	*E. coli*	x	x	x						x			x	x	-	nt	A, M	-	Ic	-
	*E. coli*	x	x							x		x	x	x	-	-	A, B, O, M	3	Ic	-
	*E. coli*	x	x		x	x	x	x		x			x	x	-	nt	K	1, 3	Ia	1
	*E. coli*	x	x		x		x	x		x			x	x	-	nt	A	2, 3	IIb	1
	*E. coli*	x	x					x		x	x	x	x	x	I	-	A, M	2, 3	Ic, IIb	1
	*S. enterica*	x						x		x			x		-	nt	-	3	Ia, IIIb	2
	*E. vulneris*	x								x	x		x	x	-	-	B, M	1, 2, 3	Ic, IIb	-
	*Enterobacter cloacae*	x	x	x	x			x							-	nt	-	-	-	-
	*C. freundii*	x	x	x	x	x		x					x		-	nt	-	3	IIIb	-
	nt	x						x						x	-	nt	A(P)	-	-	1
	nt	x								x	x			x	-	B, S	A, M	3	-	-
	nt	x								x	x		x	x	-	B, S	B	3	-	-
	nt	x						x		x			x	x	-	nt	A, A(P), M	3	IIIb	2
	nt	x	x							x			x	x	-	nt	A	1	-	-

^(a)^ Isolates origin per enterprise type: Poultry, Pig, Dairy and Slaughterhouse; ^(b)^ AB (antibiotics): AMC, amoxicillin/clavulanic acid; FOX, cefoxitin; CTX, cefotaxime; CAZ, ceftazidime; CPO, cefpirome; ATM, aztreonam; IPM, imipenem; MEM, meropenem; CIP, ciprofloxacin; GEN, gentamicin; CHL, chloramphenicol; SXT, trimethoprim/sulfamethoxazol; TET, tetracycline; nt: not tested; -: gene not present.

**Table 3:** Absolute and relative frequency of isolates with *cat*, *qnr*, *oqx*, *aac*(6’)-Ib, *qep*, *tet*, *sul*, *dfr* and *intI* genes per enterprise type.

Target Gene/Group		Enterprise Type				Total *
		Poultry *	Pig *	Dairy *	Slaughterhouse *	
*cat*	I	8 (36.4)	3 (15.7)	3 (23.1)	1 (6.3)	15 (21.4)
	II			2 (15.4)		2 (2.9)
	III, IV					0 (0.0)
*qnr*	A					0 (0.0)
	B	4 (30.8)	6 (50.0)		2 (40.0)	12 (36.4)
	C	4 (30.8)		2 (66.7)		6 (18.1)
	D	2 (15.4)				2 (6.1)
	S	11 (84.6)	2 (16.7)	3 (100.0)	2 (40.0)	18 (54.6)
*oqx*	A					0 (0.0)
	B		2 (16.7)			2 (6.1)
*aac*(6’)-Ib						0 (0.0)
*qep*	A					0 (0.0)
*tet*	A	19 (86.4)	15 (78.9)	9 (69.2)	7 (43.8)	50 (71.4)
	B	1 (4.6)	2 (10.5)		3 (18.8)	6 (8.6)
	C	3 (13.6)				3 (4.3)
	E	2 (9.1)		3 (23.1)		5 (7.1)
	K	4 (18.2)	1 (5.3)	1 (7.7)	1 (6.3)	7 (10.0)
	L	5 (22.7)			1 (6.3)	6 (8.6)
	M	4 (18.2)	5 (26.3)	1 (7.7)	7 (43.8)	17 (24.3)
	O	1 (4.6)		1 (7.7)	2 (12.5)	4 (5.7)
	A(P)	2 (9.1)			3 (18.8)	5 (7.1)
	D, G, S, Q, X					0 (0.0)
*sul*	1	15 (68.2)	5 (26.3)	7 (53.8)	3 (18.8)	30 (42.9)
	2	9 (40.9)	11 (57.9)	4 (30.8)	3 (18.8)	27 (38.6)
	3	15 (68.2)	9 (47.4)	4 (30.8)	11 (68.8)	40 (57.1)
*dfr*	Ia	17 (77.2)	9 (47.4)	6 (46.2)	4 (25.0)	36 (51.4)
	Ib	4 (18.2)		3 (23.1)		7 (10.0)
	Ic	5 (22.7)	5 (26.3)		5 (31.3)	15 (21.4)
	IIa	1 (4.6)	2 (10.5)	1 (7.7)		4 (5.7)
	IIb	6 (27.3)			3 (18.8)	9 (12.9)
	IIc			1 (7.7)		1 (1.4)
	IIIa	3 (13.6)				3 (4.3)
	IIIb		4 (21.1)	2 (15.4)	3 (18.8)	9 (12.9)
	IIIc	2 (9.1)		2 (15.4)		4 (5.7)
	IVa, IVb, IVc					0 (0.0)
	Va, Vb, Vc, Vd					0 (0.0)
*intI*	1	5 (22.7)	5 (26.3)	2 (15.4)	4 (25.0)	16 (22.9)
	2		6 (31.6)		3 (18.8)	9 (12.9)
	3					0 (0.0)

* n (%), absolute and (relative) frequency of isolates carrying the gene. The blank space means a negative result, i.e., 0 (0%).

**Table 4:** Resistance phenotype of isolates from four livestock enterprises to non-β-lactamic AB and corresponding presence of integrons class.

AB	Resistant Isolates (N)	Resistant Isolates with Integrons, N (%)		Resistant Isolates without Integrons, N (%)
		*intI*1	*intI*2	
CHL	58	14 (24.1%)	8 (13.8%)	36 (62.1%)
CIP	29	10 (34.5%)	1 (3.4%)	18 (62.1%)
GEN	7	1 (14.3%)	0 (0.0%)	6 (85.7%)
SXT	56	15 (26.8%)	8 (14.3%)	33 (58.9%)
TET	63	13 (20.6%)	8 (12.7%)	42 (66.7%)

N (%), absolute and (relative) frequency of isolates carrying the gene.

## Supplementary Materials

The following are available online at https://www.mdpi.com/2079-6382/8/1/23/s1, Figure S1: Relative frequency of antibiotics resistance by species. Table S1: Primers used for the identification of chloramphenicol (*cat*), trimethoprim (*dfr*), quinolones (*qnr*, *aac*(6’)-Ib, *oqx*, *qep*), sulphonamides (*sul*) and tetracyclines (*tet*) resistance genes and for integron class, Table S2: Multiplex PCR conditions for target genes.
