# Supplementary material for: Prevalence of Antibiotic Resistance Genes in Multidrug-Resistant Enterobacteriaceae on Portuguese Livestock Manure
Source: Antibiotics (Basel). 2019 Mar 13;8(1):23. doi: 10.3390/antibiotics8010023 (PMC6466527; doi:10.3390/antibiotics8010023)
Supplement: Supplementary file 1 [file antibiotics-08-00023-s001.pdf]

antibiotics

Article

Prevalence of Antibiotic Resistance Genes in Multidrug-Resistant *Enterobacteriaceae* on Portuguese Livestock Manure

Paula Amador [1,*], Ruben Fernandes [2], Cristina Prudêncio [2] and Isabel Duarte [1]

[1] Environment Department, Research Centre for Natural Resources, Environment and Society (CERNAS), College of Agriculture, Polytechnic of Coimbra, 3045-601 Coimbra, Portugal; iduarte@esac.pt
[2] Department Chemical Sciences and Biomolecules, School Allied Health Sciences, Polytechnic of Porto, 4200-072 Porto, Portugal; rfernandes@ess.ipp.pt (R.F.); cprudencio@estsp.ipp.pt (C.P.)
* Correspondence: paula_amador@esac.pt; Tel.: +351-129-802-940

Supplementary Materials

Table S1. Primers used for the identification of chloramphenicol (*cat*), trimethoprim (*dfr*), quinolones (*qnr, aac*(6´)-Ib, *oqx, qep*), sulphonamides (*sul*) and tetracyclines (*tet*) resistance genes and for integron class.

Target gene/group	Primers sequences (5´-3´) Fw/Rv	Amplicon size (bp)	Primers (µM)	Reference
cat I	GGTGATATGGGATAGTGTT/CCATCACATACTGCATGATG	349	1.0	[1]
cat II	GATTGACCTGAATACCTGGAA/CCATCACATACTGCATGATG	567	1.0	[1]
cat III	CCATACTCATCCGATATTGA/CCATCACATACTGCATGATG	275	1.0	[1]
cat IV	CCGGTAAAGCGAAATTGTAT/CCATCACATACTGCATGATG	451	1.0	[1]
dfr Ia	GTGAAACTATCACTAATGG/ACCCTTTTGCCAGATTTG	471	1.0	[2]
dfr Ib	TTGGGAAGGACAACGCACTT/ACCATTTCGGCCAGATCAAC	382	1.0	[3]
dfr Ic	GGTGAGCARAAGATYTTTCGC/TGGGAAGAAGGCGTCACCCTC	309	1.0	[2]
dfr IIa	GCBAAAGGDGARCAGCT/TTTMCCAYATTTGATAGC	394	1.0	[2]
dfr IIb	AAAATTTCATTGATTTCTGCA/TTAGCCTTTTTTCCAAATCT	471	1.0	[2]
dfr IIc	TTTATTGTGGTAAGCAATAC/GTATACATCTGCATCAAAAC	201	1.0	[2]
dfr IIIa	ACCTGCCGATCTGCGTCAT/TCGCAGGCATAGCTGTTCTT	387	1.0	[3]
dfr IIIb	ACCAGAGCATTCGGTAATCA/TTGGATCACCTACCCATAGA	445	1.0	[3]
dfr IIIc	CACAGTCTATCGCCTTAATC/ATAGACCACAAAGCTAAACG	233	1.0	[2]
dfr IVa	GTTTCCGAGAATGGAGTAAT/GGTACGTGTAATCAATATTTG	429	1.0	[3]
dfr IVb	TCACCAAGAAGTCAGAGATT/TAAAACCAGATTCGACTTTC	311	1.0	[2]
dfr IVc	AGAATTCCCTTCTCTTTGAT/ATGCCAACAGTTGAGATTAT	218	1.0	[2]
dfr Va	GATCACGTRCGCAAGAARTC/GACTCGACVGCRTASCCTTC	95	1.0	[2]
dfr Vb	TGAACCAGAAGATTTAAAACAC/AATGGTCGGGACCTCAGAT	384	1.0	[2]
dfr Vc	AGTCGCTGTGGATTCTAAGT/CAATGTGAAAATTGTTCTGG	455	1.0	[2]
dfr Vd	ATGATTTGCTTTGGCACTTA/CCACCAATAATGAAGCATGT	250	1.0	[2]
*sul*1	CGGCGTGGGCTACCTGAACG/GCCGATCGCGTGAAGTTCCG	433	0.4	[4]
*sul*2	GCGCTCAAGGCAGATGGCATT/GCGTTTGATACCGGCTCCCGT	293	0.4	[4]
*sul*3	GAGCAAGATTTTGGAATCG/CATCTGCAGCTAACCTAGGGCTTTGGA	790	0.4	[5]
tet(A)	GCTACATCCTGCTTGCCTTC/CATAGATCGCCGTGAAGAGG	210	1.0	[6]
tet(B)	TTGGTTAGGGGCAAGTTTTG/GTAATGGGCCAATAACACCG	659	0.25	[6]
tet(C)	CTTGAGAGCCTTCAACCCAG/ATGGTCGTCATCTACCTGCC	418	0.25	[6]
tet(D)	AAACCATTACGGCATTCTGC/GACCGGATACACCATCCATC	787	2.0	[6]
tet(E)	AAACCACATCCTCCATACGC/AAATAGGCCACAACCGTCAG	278	1.0	[6]
tet(G)	CAGCTTTCGGATTCTTACGG/GATTGGTGAGGCTCGTTAGC	468	1.0	[6]
tet(K)	TCGATAGGAACAGCAGTA/CAGCAGATCCTACTCCTT	844	1.25	[6]
tet(L)	TCGTTAGCGTGCTGTCATTC/GTATCCCACCAATGTAGCCG	267	1.0	[6]

Target gene/group	Primers sequences (5′-3′) Fw/Rv	Amplicon size (bp)	Primers (μM)	Reference
***tet*(M)**	GTGGACAAAGGTACAACGAG/CGGTAAAGTTCGTCACACAC	406	0.5	[6]
***tet*(O)**	AACTTAGGCATTCTGGCTCAC/TCCCACTGTTCCATATCGTCA	515	1.25	[6]
***tet*(S)**	CATAGACAAGCCGTTGACC/ATGTTTTTGGAACGCCAGAG	667	0.5	[6]
***tet*A(P)**	CTTGGATTGCGGAAGAAGAG/ATATGCCCATTTAACCACGC	676	1.25	[6]
***tet*(Q)**	TTATACTTCCTCCGGCATCG/ATCGGTTCGAGAATGTCCAC	904	1.25	[6]
***tet*(X)**	CAATAATTGGTGGTGGACCC/TTCTTACCTTGGACATCCCG	468	1.25	[6]
***qnr* A**	AGAGGATTTCTCACGCCAGG/TGCCAGGCACAGATCTTGAC	580	0.25	[7]
***qnr* B**	GGMATHGAAATTCGCCACTG/TTTGCYGYYCGCCAGTCGAA	264	0.25	[7]
***qnr* C**	GGGTTGTACATTTATTGAATCG/CACCTACCCATTTATTTTCA	307	0.25	[8]
***qnr* D**	CGAGATCAATTTACGGGGAATA/AACAAGCTGAAGCGCCTG	465	0.25	[9]
***qnr* S**	GCAAGTTCATTGAACAGGGT/TCTAAACCGTCGAGTTCGGCG	428	0.25	[7]
***aac*(6′)-Ib**	TTGCGATGCTCTATGAGTGGCTA/CTCGAATGCCTGGCGTGTTT	482	0.25	[10]
***oqx* A**	CTCGGCGCGATGATGCT/CCACTCTTCACGGGAGACGA	392	0.25	[8]
***oqx* B**	TCCTGATCTCCATTAACGCCCA/ACCGGAACCCATCTCGATGC	131	0.25	[8]
***qep* A**	CCAGCTCGGCAACTTGATAC/ATGCTCGCCTTCCAGAAAA	570	0.25	[11]
***intI*1**	GGGTCAAGGATCTGGATTTCG/ACATGCGTGTAAATCATCGTCG	465	0.3	[12]
***intI*2**	CACGGATATGCGACAAAAAGGT/GTAGCAAACGAGTGACGAAATG	788	0.3	[12]
***intI*3**	GCCTCCGGCAGCGACTTTCAG/ACGGATCTGCCAAACCTGACT	979	0.3	[12]

Table S2. Multiplex PCR conditions for target genes.

Target gene/group		Cycling conditions						Final concentrations		
		1st step[a]	Cycles	2nd step[b]	3rd step[c]	4th step[d]	5th step[e]	$MgCl_2$ (mM)	dNTP (μM)	*Taq* pol. (U)
cat	**I, II, III, IV**	95/3′	34x	95/60″	55/60″	72/90″	72/5′	3.0	300	1.5
intI	***1, 2, 3***	94/5′	30x	94/30″	62/30″	72/60″	72/8′	5.0	360	1.0
sul	**1, 2**	94/5′	30x	94/15″	69/30″	72/60″	72/7′	2.0	200	0.5
sul	**3**				51/30″					
tet	**A, E, G, K, L, M, O, S**	94/5′	35x	94/60″	55/60″	72/90″	72/7′	3.0	300	2.5
tet	**B, C, D, A(P), Q, X**							4.0		
dfr	**I**	94/2′	30x	94/30″	46/30″	72/30″	72/1′	2.5	200	1.0
dfr	**II**				44/30″					
dfr	**III, IV**				52/30″					
dfr	**V**				45/30″					
qnr	**A, B**	95/5′	35x	95/45″	60/45″	72/60″	72/10′	2.5	300	1.0
aac	**(6′)-Ib-cr**									
qnr	**C, D**				52/45″				200	
qep	**A**									
qnr	**S**				54/45″				300	
oqx	**A**									
oqx	**B**				62/45″					

a) 1st denaturation, b) 2nd denaturation, c) annealing, d) extension, e) final extension, all expressed with temperature in °C /time in minutes, ′ or seconds, ″.

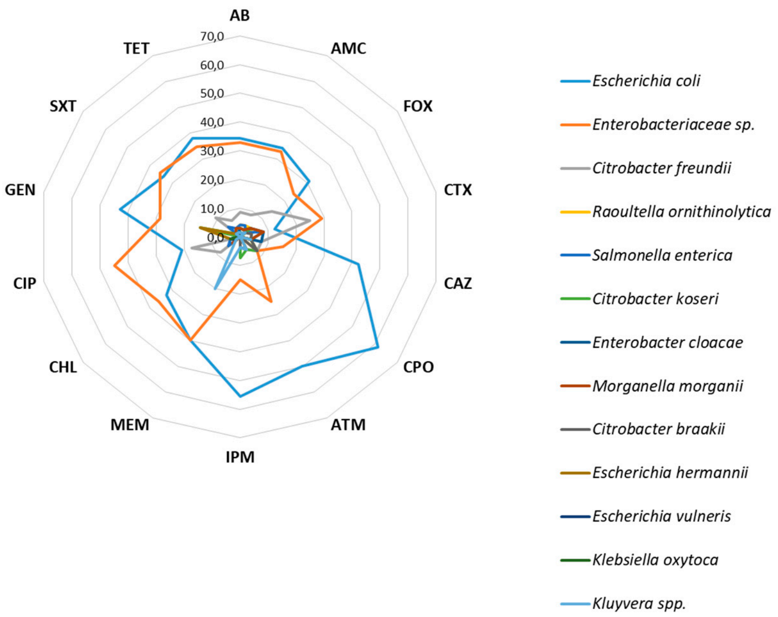

Figure S1. Relative frequency of antibiotics resistance by species.

References

1. Ng, K.H.; Samuel, L.; Kathleen, M.M.; Leong, S.S.; Felecia, C. Distribution and prevalence of chloramphenicol-resistance gene in *Escherichia coli* isolated from aquaculture and other environment. *Int. Food Res. J.* **2014**, *21*, 1321–1325.
2. Šeputienė, V.; Povilonis, J.; Ružauskas, M.; Pavilonis, A.; Sužiedėlienė, E. Prevalence of trimethoprim resistance genes in *Escherichia coli* isolates of human and animal origin in Lithuania. *J. Med. Microbiol.* **2010**, *59*, 315–322, doi:10.1099/jmm.0.015008-0.
3. Chen, S.; Zhao, S.; White, D.G.; Schroeder, C.M.; Lu, R.; Yang, H.; McDermott, P.F.; Ayers, S.; Meng, J. Characterization of multiple-antimicrobial-resistant *Salmonella* Serovars isolated from retail meats. *Appl. Environ. Microb*. **2004**, *70*, 1–7, doi:10.1128/AEM.70.1.1–7.2004.
4. Kerrn, M.B.; Klemmensen, T.; Frimodt-Møller, N.; Espersen, F. Susceptibility of Danish *Escherichia coli* strains isolated from urinary tract infections and bacteraemia and distribution of *sul* genes conferring sulphonamide resistance. *J. Antimicrob. Chemother.* **2002**, *50*, 513–516, doi:10.1093/jac/dkf164.
5. Perreten, V.; Boerlin, P. A new sulfonamide resistance gene (*sul*3) in *Escherichia coli* is widespread in the pig population of Switzerland. *Antimicrob. Agents Chemother.* **2003**, *47*, 1169–1172, doi:10.1128/AAC.47.3.1169–1172.2003.
6. Ng, L.-K.; Martin, I.; Alfa, M.; Mulvey, M. Multiplex PCR for the detection of tetracycline resistant genes. *Mol. Cell. Probes* **2001**, *15*, 209–215, doi:10.1006/mcpr.2001.0363.
7. Cattoir, V.; Poirel, L.; Rotimi, V.; Soussy, C.J.; Nordmann, P. Multiplex PCR for detection of plasmid-mediated quinolone resistance *qnr* genes in ESBL-producing enterobacterial isolates. *J. Antimicrob. Chemother.* **2007**, *60*, 394–397, doi:10.1093/jac/dkm204.
8. Kim, H.B.; Wang, M.; Park, C.H.; Kim, E.C.; Jacoby, G.A.; Hooper, D.C. *oqx*AB encoding a multidrug efflux pump in human clinical isolates of *Enterobacteriaceae*. *Antimicrob. Agents Chemother.* **2009**, *53*, 3582–3584, doi:10.1128/AAC.01574-08.
9. Cavaco, L.M.; Hasman, H.; Xia, S.; Aarestrup, F.M. *qnr*D, a novel gene conferring transferable quinolone resistance in *Salmonella enterica* Serovar Kentucky and Bovismorbificans strains of human origin. *Antimicrob. Agents Chemother*. **2009**, *53*, 603–608, doi:10.1128/AAC.00997-08.
10. Park, C.H.; Robicsek, A.; Jacoby, G.A.; Sahm, D.; Hooper, D.C. Prevalence in the United States of *aac*(6')-Ib-cr encoding a ciprofloxacin-modifying enzyme. *Antimicrob. Agents Chemother.* **2006**, *50*, 3953–3955, doi:10.1128/AAC.00915-06.

11. Xia, L.N.; Li, L.; Wu, C.M.; Liu, Y.Q.; Tao, X.Q.; Dai, L.; Qi, Y.H.; Lu, L.M.; Shen, J.Z. A survey of plasmid-mediated fluoroquinolone resistance genes from *Escherichia coli* isolates and their dissemination in Shandong, China. *Foodborne Pathog. Dis.* **2010**, *7*, 207–215, doi:10.1089/fpd.2009.0378.
12. Mazel, D.; Dychinco, B.; Webb, V.A.; Davies, J. Antibiotic resistance in the ECOR collection: Integrons and identification of a novel *aad* gene. *Antimicrob. Agents Chemother.* **2000**, *44*, 1568–1574.

© 2019 by the authors. Submitted for possible open access publication under the terms and conditions of the Creative Commons Attribution (CC BY) license (http://creativecommons.org/licenses/by/4.0/).
